# Supplementary material for: Investigating the influence of physiologically relevant hydrostatic pressure on CHO cell batch culture
Source: Sci Rep. 2021 Jan 8;11:162. doi: 10.1038/s41598-020-80576-8 (PMC7794228; doi:10.1038/s41598-020-80576-8)
Supplement: Supplementary file 1 — Supplementary Information 1. [file 41598_2020_80576_MOESM1_ESM.zip › S1/IgG/Feb14 0 30 60 90/60mmHg Jan30.pdf]

===

Acq. Operator : SYSTEM Seq. Line : 14
Sample Operator : SYSTEM
Acq. Instrument : HPLC-0039 Location : 8
Injection Date : 2/14/2019 11:50:16 AM Inj : 1
Inj Volume : 100.000 µl
Method : C:\Users\Public\Documents\ChemStation\1\Data\TK_CHO_IgG 2019-02-14 10-52-34
\CHO_IgG_LC.M (Sequence Method)
Last changed : 2/8/2019 3:17:23 PM by SYSTEM

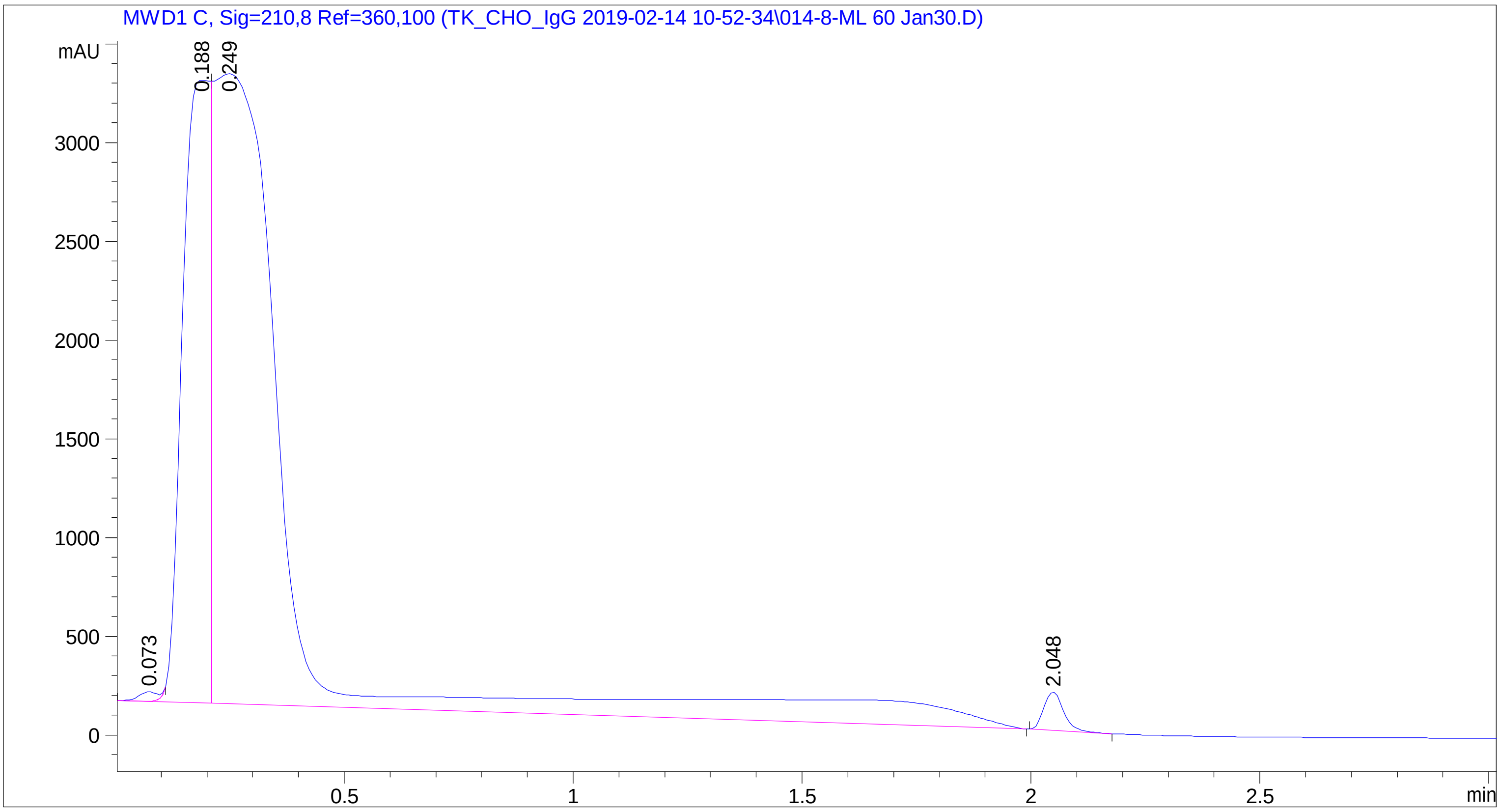

===

Area Percent Report

===

Sorted By : Signal
Multiplier : 1.0000
Dilution : 1.0000
Use Multiplier & Dilution Factor with ISTDs

Signal 1: MWD1 C, Sig=210,8 Ref=360,100

Peak #	RetTime [min]	Type	Width [min]	Area [mAU*s]	Height [mAU]	Area %
1	0.073	BV E	0.0422	128.29192	46.49269	0.2614
2	0.188	VV R	0.0637	1.28885e4	3152.64136	26.2612
3	0.249	VB	0.1657	3.54780e4	3190.73267	72.2890
4	2.048	BBA	0.0472	583.19513	193.52156	1.1883

Totals : 4.90780e4 6583.38828

===

*** End of Report ***
